# Exploring the Role of the Extracellular Matrix in Disease and Aging

**DOI:** 10.14789/ejmj.JMJ25-0033-P

**Published:** 2025-12-18

**Authors:** ERI ARIKAWA-HIRASAWA

**Affiliations:** 1 Research Institute for Diseases of Old Age, Graduate School of Medicine, Juntendo University, Tokyo, Japan; 1 Research Institute for Diseases of Old Age, Graduate School of Medicine, Juntendo University, Tokyo, Japan

**Keywords:** extracellular matrix, aging, muscular dystrophy, laminin, perlecan

## Abstract

Throughout my research career, I have focused on understanding the extracellular matrix (ECM) and its roles in disease pathogenesis and the aging process. My initial interest in muscular dystrophies gradually expanded to include various organs and systems, including the kidneys, brain, cartilage, skin, and eyes. During my tenure at the National Institutes of Health (NIH), I made substantial contributions to generating and analyzing conditional knockout mouse models for key ECM molecules, including laminin α1 and perlecan. These studies elucidated the roles of ECM components in hereditary diseases, embryonic development, and the functionality of the neuromuscular junction. Upon returning to Japan, I transitioned this foundational expertise into translational research across multiple fields, benefiting from a collaborative environment that bridges basic and clinical sciences. My recent work examines the impact of glycosylation on ECM remodeling. Reflecting on this scientific journey, I emphasize the importance of ECM as a structural component and as a dynamic regulator of cellular behavior. As I retire from active academic life, I aspire to support the next generation of scientists in exploring the extracellular space, an area rich with potential therapeutic opportunities.

Upon graduating from Juntendo University in 1984, I began my residency in the Department of Neurology. Although my clinical duties primarily involved treating patients with Parkinson’s disease, I was greatly influenced by the research of the late Dr. Kiichi Arahata (Class of 1971) in neuromuscular diseases and neuroimmunology. This influence prompted my transition from clinical practice to basic research on muscle diseases under the supervision of Dr. Arahata at the National Institute of Neuroscience, National Center of Neurology and Psychiatry. Around that time, it became more evident that muscular dystrophies, long considered intractable, originated from disruptions in linkages between the interior and exterior environment of cells. Consequently, many causative genes were identified. Being involved in this dynamic research area, I became interested in the cellular microenvironment, which motivated me to pursue a research career focusing on the ECM.

## Research at the NIH in the United States

**Figure 1:**
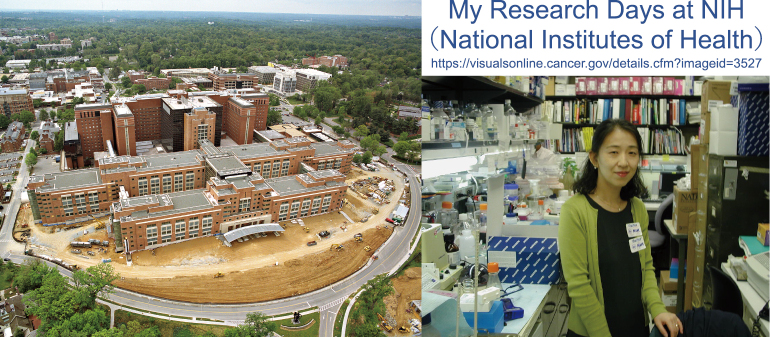
View of the main campus of the National Institutes of Health (NIH) in Bethesda, Maryland, and a snapshot taken in the laboratory

**Figure 2:**
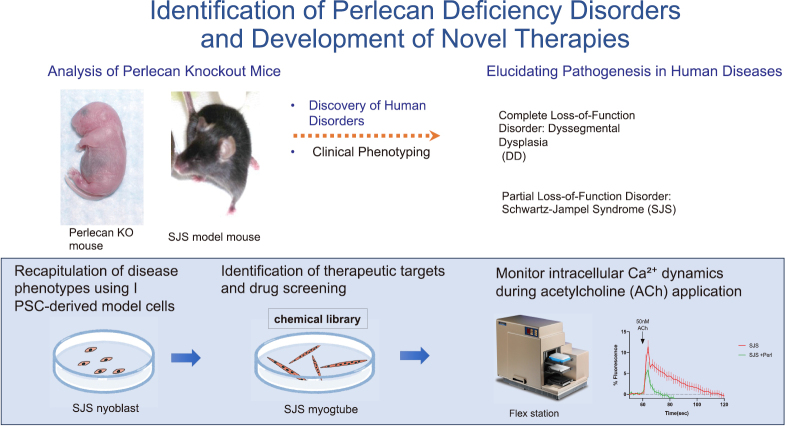
Identification of Perlecan Deficiency Disorders and Development of Novel Therapies. Analysis of Perlecan knockout mice led to the identification of human disorders, including dyssegmental dysplasia (DD) and Schwartz–Jampel syndrome (SJS), providing insights into disease mechanisms and therapeutic development.

After completing my tenure as a chief resident, I gave birth to my child, and then I pursued training at the National Institutes of Health (NIH) in the United States (Figure 1), a global hub for ECM research. During five years of dedicated research, I developed skills in key cell culture and molecular biology techniques, generating and analyzing conditional knockout mice for two critical basement membrane components: laminin α1 and the heparan sulfate proteoglycan perlecan. Through extensive analysis of the perlecan knockout mice, we demonstrated that the loss of perlecan is the pathogenic basis for two distinct hereditary diseases (Figure 2), significantly contributing to our understanding of their mechanisms. We identified novel roles for perlecan at the neuromuscular junction. Consequently, I published four^1)^ first-author publications in high-impact journals^1-4)^. Despite the time constraints of raising a child, this period was the most productive phase of my academic career, underscoring the importance of a highly supportive and focused research environment.

## Translating ECM research to clinical applications

Upon returning to Japan, I began translating my ECM research experience into a clinical context at Juntendo University, where collaboration between basic and clinical researchers is strongly encouraged to bridge this gap. This framework led to the expansion of my ECM research beyond the neuromuscular system to include organs such as the kidney, cartilage, skin, and eye. Much of the analysis of the laminin α1 knockout mouse was conducted after returning to Japan, and they elucidated its roles in embryonic, renal, and brain development. Furthermore, through the analysis of these two ECM molecules, my research continued to explore the role of ECM in both disease pathogenesis and the aging process. Currently, my research focuses on two primary areas: investigating how glycosylation modulates ECM function and characterizing ECM remodeling^5-11)^.

## Reflection on my research journey

My research journey began with muscular dystrophy, then expanded into the field of extracellular biology, and I advanced my research using gene modification technologies. I have identified promising potential for developing new therapies by elucidating how ECM contributes to disease and aging. As part of efforts to translate these findings to the benefit of patients with rare and intractable diseases, I now serve on the Tokyo Metropolitan Government's Intractable Disease Review Board.

As I reach retirement and reflect on my journey as a researcher since graduating from Juntendo University, I am filled with deep gratitude for the mentors, colleagues, juniors, and students who supported me along the way. The ECM, which dynamically interacts with various cells, regulating their function and directing their fate while continuously transforming itself, has mirrored my own collaborative research career path at Juntendo University.

Going forward, I aim to utilize my experience to promote greater diversity and foster supportive environments where female and young researchers can thrive. I hope that the next generation of researchers will continue to pay more attention to the world outside the cell and explore the vital roles of the extracellular matrix, leading to novel discoveries.

## Author contributions

EAH analyzed and interpreted the literature and data across ECM-related systems, drafted and revised the manuscript, and approved the final version.

## Conflicts of interest statement

The author declare that there are no conflicts of interest. Eri Arikawa-Hirasawa, one of the Editorial Board members of JMJ was not involved in the peer review or decision-making process for this paper.
